# Supplementary material for: Attosecond nanoscale near-field sampling
Source: Nat Commun. 2016 May 31;7:11717. doi: 10.1038/ncomms11717 (PMC4895016; doi:10.1038/ncomms11717)
Supplement: Supplementary Information — Supplementary Figures 1-12, Supplementary Notes 1-9 and Supplementary References [file ncomms11717-s1.pdf]

Supplementary Figure 1

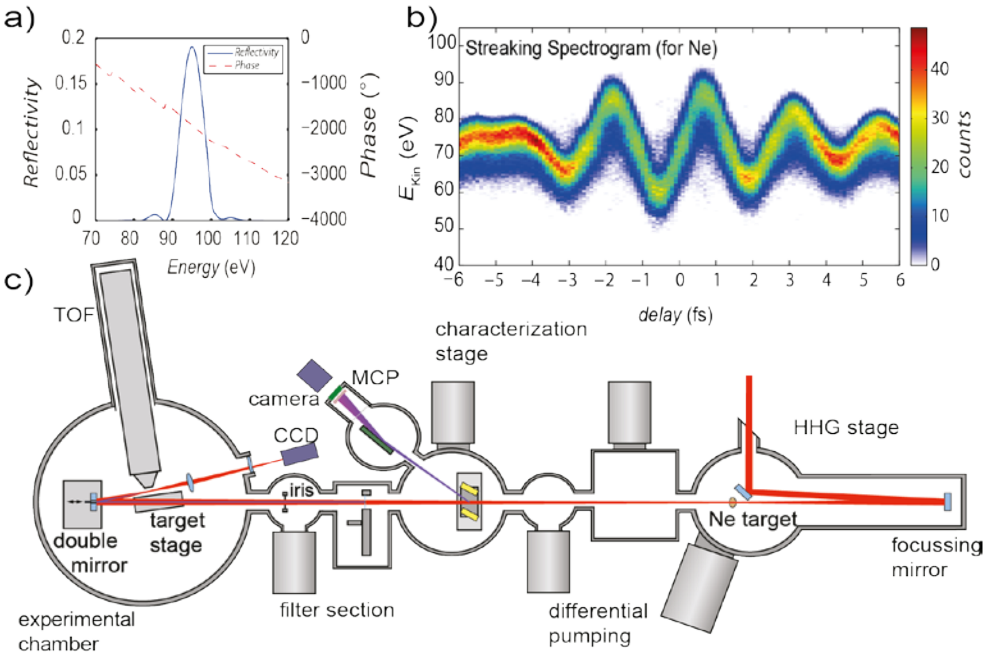

Schematic of the experimental setup. The attosecond beamline AS-5 as sketched in (c) consists of four major parts, i.e. HHG stage, characterization stage, filter section and experimental chamber. Few-cycle pulses enter the beamline through a 1 mm thick Brewster-angle window and are focused into a Ne gas target for high-harmonic generation using a spherical mirror with focal length of 50 cm. After a subsequent differential pumping stage the harmonics spectrum can be characterized in terms of spectral intensity and beam profile by deflecting the beam utilizing flat gold-coated mirrors that can be positioned via a motorized stage. Spectral filtering of the XUV spectrum and spatial separation of XUV and NIR pulses is accomplished using a filter consisting of a 3 mm diameter Zr-foil mounted in the center of a nitrocellulose pellicle. An adjustable iris allows setting the NIR intensity impinging on the target. In the experimental chamber the two beams are focused independently by a double mirror assembly with an optimized reflectivity for the XUV pulse (a). The multilayer XUV mirror is designed such that a single attosecond pulse can be extracted at 95 eV. By focusing the two beams into a jet of Ne gas we measure a streaking spectrogram (b), permitting to extract the vector potential of the few-cycle light field and the attosecond pulse properties.

Supplementary Figure 2

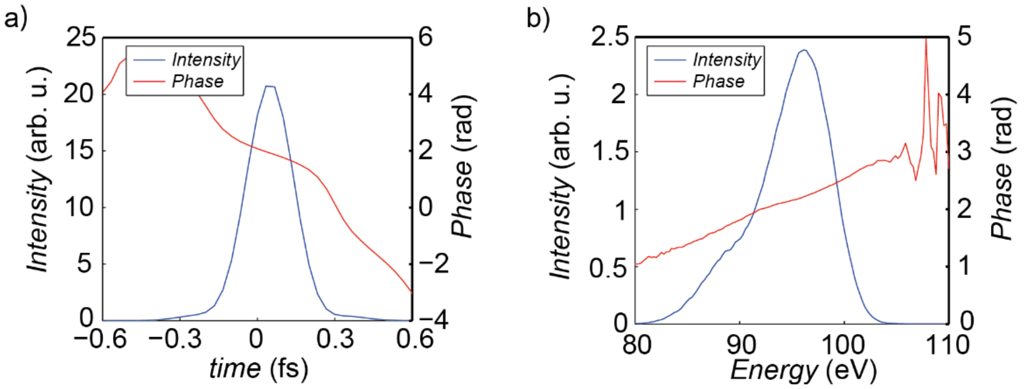

Attosecond pulse properties. Duration (a) and spectrum (b) of the attosecond laser pulse retrieved using an iterative FROG-CRAB algorithm (ATTOGRAM)[4]. As input of the retrieval process, we use the measured streaking curve shown in Supplementary Fig. 1(b).

Supplementary Figure 3

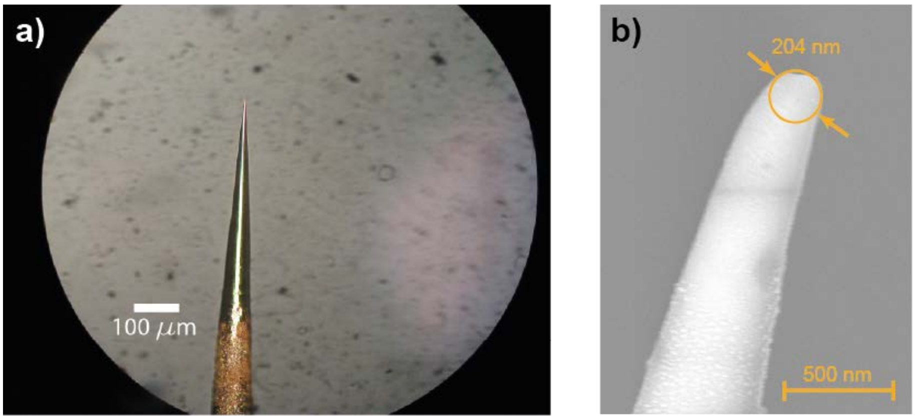

Nanotaper samples. Representative images of an etched gold nanotaper used in the attosecond streaking experiments. Coarse investigation of the samples is performed using an optical microscope (a) for a smooth and shiny surface. High resolution images taken with a scanning electron microscope show a typical apex radius of 100 nm (b).

Supplementary Figure 4

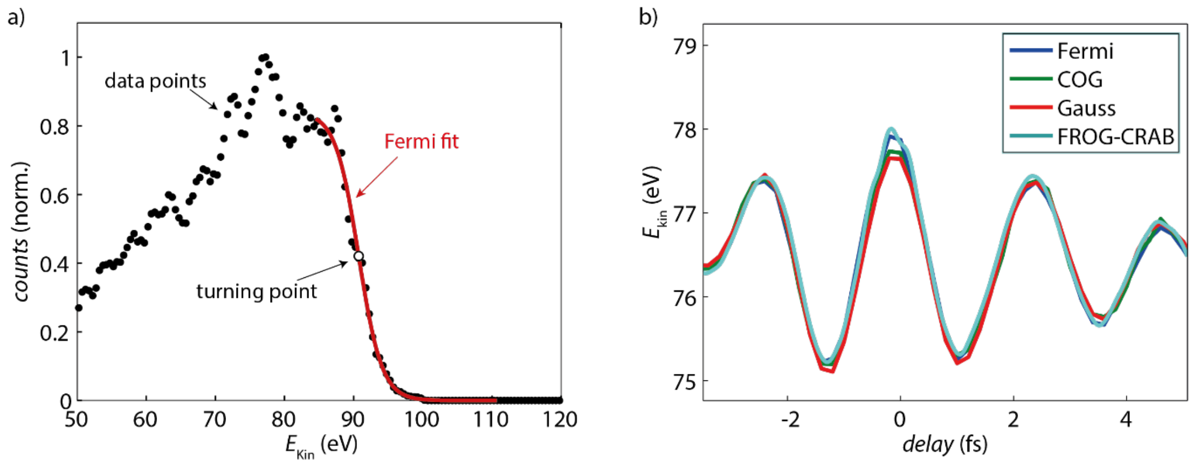

Extraction of streaking traces. (a) Illustration of the Fermi-function fit to the cut-off part of the electron kinetic energy spectrum from the nanotarget at a single delay step. The Fermi-fit is shown in red and the turning point which describes the streaking curve is indicated. (b) Comparison of the obtained streaking curves from a reference streaking spectrogram for Ne using different extraction methods, i.e. via Fermi-fit (dark blue), center-of-gravity-method (green), via Gaussian-fitting (red) and via FROG-CRAB (light blue). Good agreement is found between all curves and excellent agreement for Fermi-fit and FROG-CRAB-retrieval. The curves have been shifted to a common central energy for better comparison.

Supplementary Figure 5

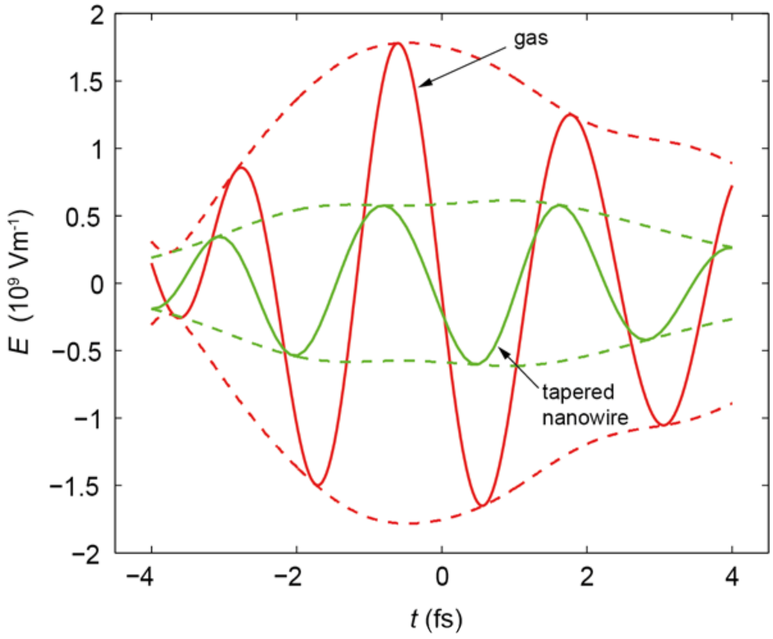

Comparison of the retrieved electric fields. Retrieved electric fields in the laser polarization direction - parallel to the tapered nanowire – for the measurement in neon (red) and on the the nanotaper (green) from the streaking traces shown in Figure 3(b) and (a), respectively. Since the response function (Fig. 4 (b) and (c)) is only slightly dependent on the energy the average relative phase and amplitude can directly be seen by comparing the two curves.

Supplementary Figure 6

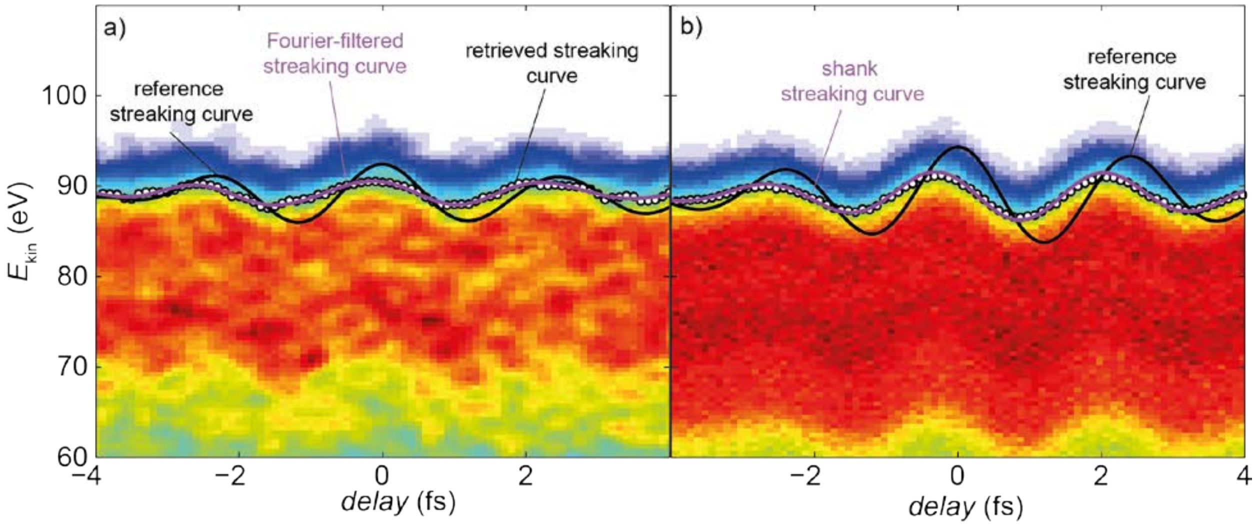

Comparison of experiment and simulation. (a) experimentally measured streaking spectrogram from the tapered nanowire together with the retrieved streaking curve via the Fermi fitting (white dots), the Fourier-filtered streaking trace (purple) and the Fourier-filtered measured incident vector potential (black line). (b) MC simulation of streaking spectrogram together with the retrieved streaking curve (white dots). Streaking curves calculated for electrons emitted from the tapered nanowire are shown as purple lines (same as in Fig. 3(d)). The reference streaking curve calculated from the vector potential of the incident laser pulse is shown as black solid line. The apex contribution is not visible in the simulated spectrogram for simulation parameters matching the experiment. From this comparison it becomes clear, that the measured streaking spectrogram is dominated by electrons emitted from the nanotaper. An input intensity of 10^{12} Wcm^{-2} has been assumed in the simulation leading to slightly higher amplitudes than observed in the experiment (see Supplementary Note 5 for further details). The statistics in the simulation is much higher; therefore the obtained spectrogram looks smoother. The reason that a clear streaking trace is obtained lies in the fact that the electric fields at the side of the tapered nanowire are relatively homogeneous and only slightly dependent on the position around the cylinder axis as well as the apex radius.

Supplementary Figure 7

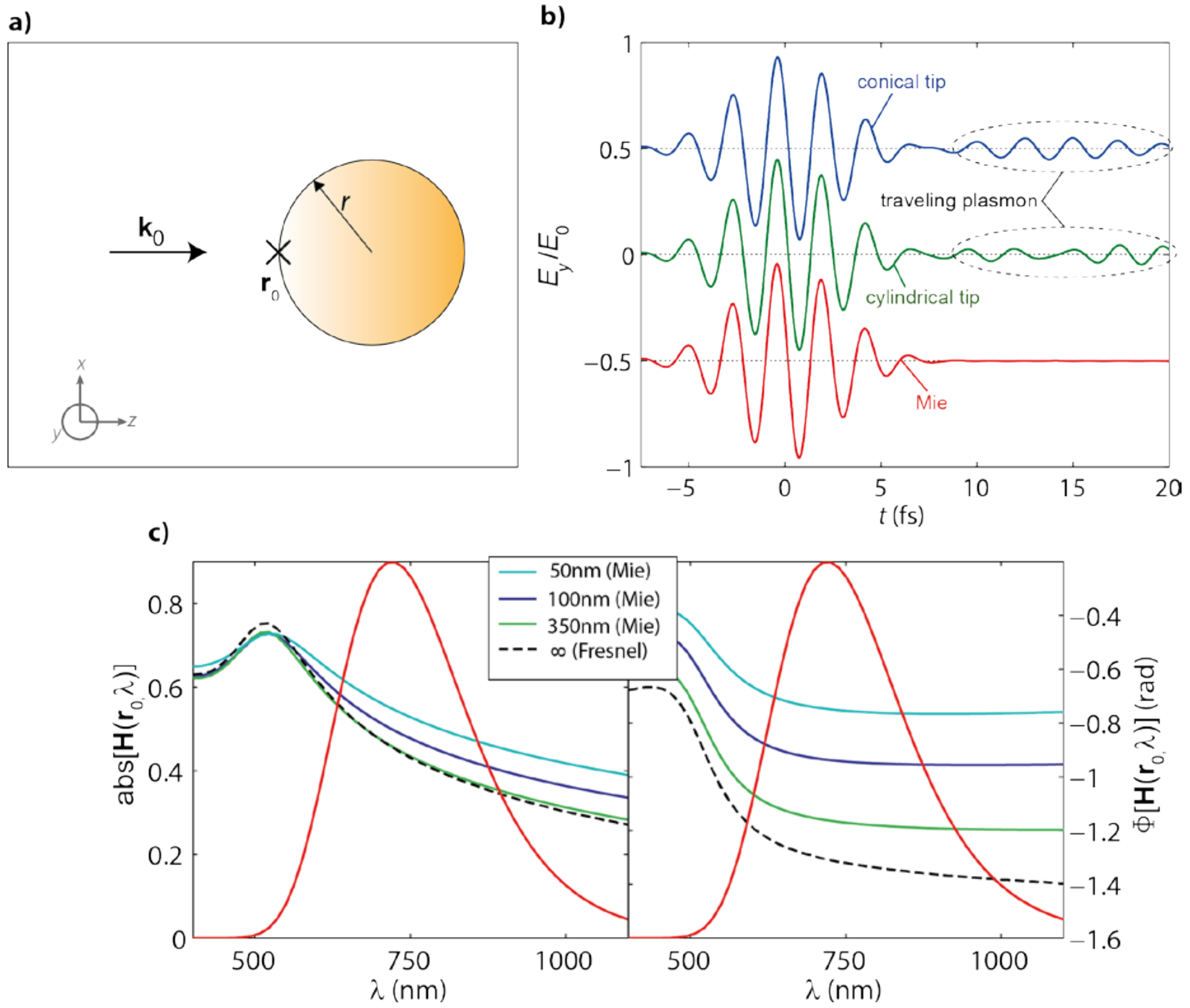

Comparison of the near-fields from the nanotapers for different geometries. (a) Illustration of the geometry, cut perpendicular to the cone-axis. The cross indicates the point where the fields are investigated. $\mathbf{k_0}$ indicates the direction of the incident pulse. The polarization is parallel to the cone/cylinder axis. (b) Comparison of the fields in the direction of the cylinder axis, for (i) a semi-infinite cylinder, terminated by a halfsphere, with 350 nm radius at a distance of 2800 nm from the apex (green), (ii) for a cone with opening angle of 12.5° and 50 nm apex radius at a distance of 2800 nm from the apex, where the distance to the cone axis (radius) is 350 nm (blue), and (iii) for an infinite cylinder with radius 350 nm (red). The blue and green lines were calculated with FDTD simulations for a Gaussian NIR beam of 15 μm diameter focused onto the apex. The red line was calculated using Mie theory[27,28] with a plane wave excitation. The blue and red curve were shifted by 0.5 up/down, respectively. The dashed ellipses indicate fields due to traveling surface plasmons excited at the apex. The refractive index was fitted to data from Ref. 10. (c) The response functions of infinite cylinders with different radii relative to the exciting pulse at the surface of the cylinder facing the exciting pulse. The shown radii are 50 nm (light blue), 100 nm (blue), 350 nm (green) and infinity, i.e. a flat surface (black dashed), which was calculated using Fresnel's equation. The red curve shows the spectral intensity of the input pulse used in (b) on a linear scale in arbitrary units.

Supplementary Figure 8

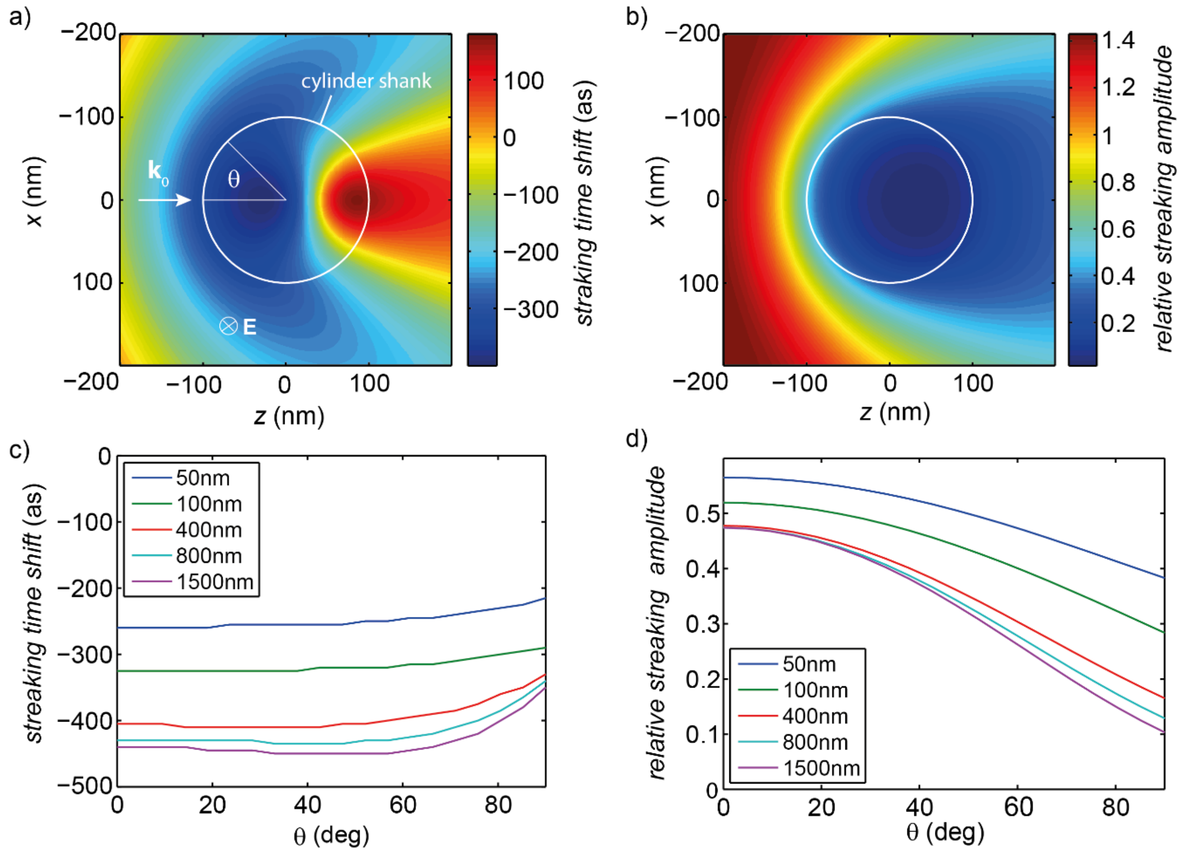

Characteristics of the vector potential (component parallel to the cylinder axis) around the gold nanotarget (cut through the cylinder). (a) The shift of the peak of the local vector potential with respect to the incoming pulse around a gold cylinder. (b) The relative amplitude of the local vector potential with respect to the incoming pulse. Electrons are only born close to the surface due to the small mean free path for inelastic scattering. (c) The shifts along the surface of the cylinder for different radii (notice the flatness of the curves). (d) The relative amplitude of the local vector potential around the cylinder for different radii. The refractive index has been taken from Palik.[30]

Supplementary Figure 9

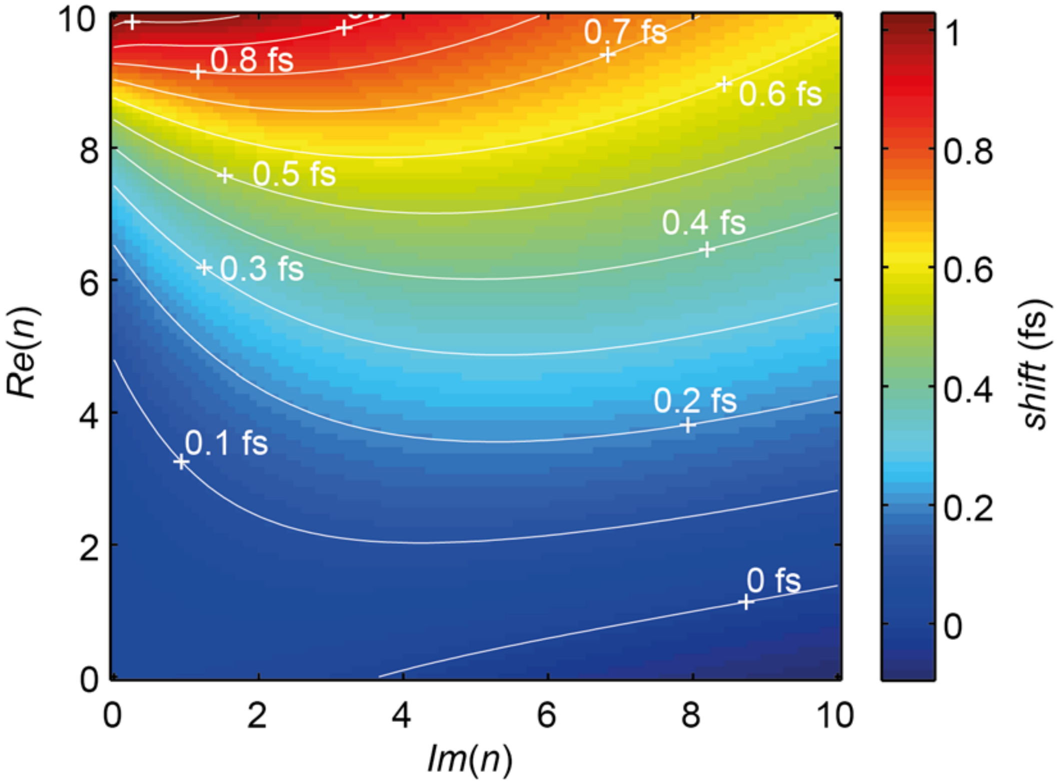

Phase shifts introduced by an adsorbate layer. The time shift of the electric field for an infinite cylinder due to an adlayer relative to an infinite cylinder without adlayer for different refractive indices: Mie calculation for the electric field component parallel to the cylinder axis, calculated for a wavelength of 720 nm, a cylinder radius of 150 nm and relatively large adlayer thickness of 5 nm.

Supplementary Figure 10

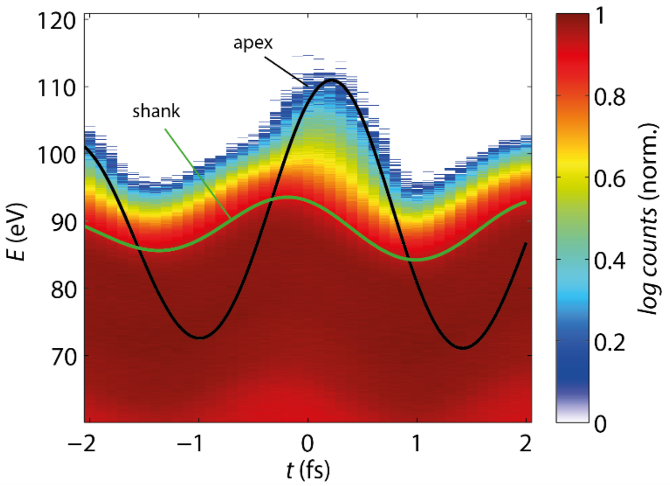

Visibility of the apex contribution in the streaking spectrogram. Simulation result with a XUV spotsize of $\mathbf{0.5\ \mu m}$ and a peak intensity of $\mathbf{3 \cdot 10^{12}\ Wcm^{-2}}$. The number of counts per delay step is $\mathbf{7 \cdot 10^{5}}$. Even for these parameters a logarithmic scale is necessary to make the apex contribution in the spectrogram visible, since the area of the enhanced region is nearly singular compared to the entire illuminated region.

Supplementary Figure 11

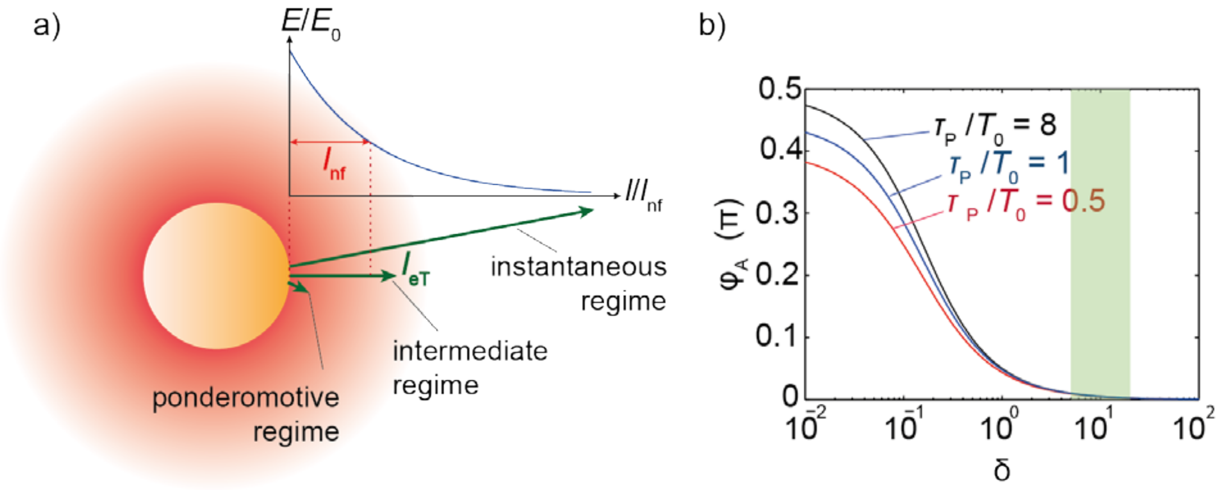

Regimes of near-field streaking. (a) Illustration of the different streaking regimes. The green arrow shows the distance an electron travels within one optical period $\boldsymbol{l_{eT}}$. While the inset shows the decay of the near-field together with the near-field decay length. (b) The shift of the streaking curve with respect to the surface vector potential calculated using eq. 15 (see Supplementary Note 9) for different ratios of δ, where $\boldsymbol{\tau_p}$ and $\boldsymbol{T_0}$ describe the temporal decay constant of the near-field and the period of the laser field, respectively. The green shaded area marks the region relevant for our experiment, which clearly is in the ponderomotive regime.

Supplementary Figure 12

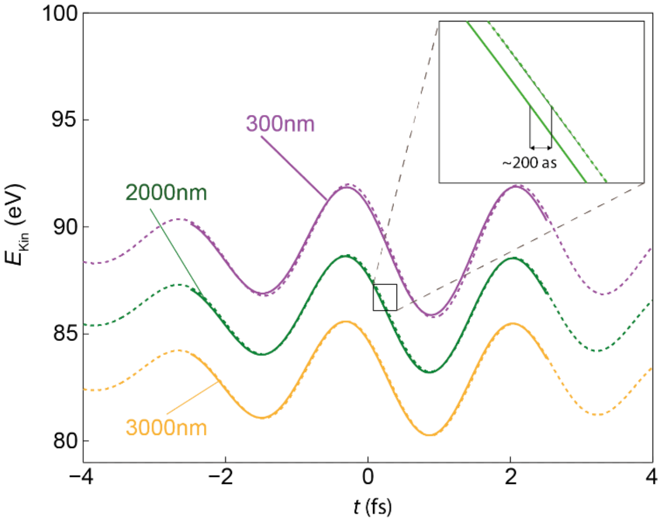

Relation of single energy streaking curves (solid lines) and local vector potential (dashed lines) for different positions on the front of the tapered nanowire. Calculation for a cylinder with 100 nm radius and 89 eV initial energy. The electrons are emitted under an angle of 15° with respect to the cylinder axis. Emission from points at a distance of 300 nm (purple), 2000 nm (green) and 3000 nm (yellow) from the apex. The green and yellow curves have been shifted down by 3 eV and 6 eV, respectively. Only a minor shift of 20 as can be observed, clearly indicating streaking in the ponderomotive regime.

Supplementary Note 1

Experimental setup

In our experiments we use the frontend of the Petawatt-Field-Synthesizer (PFS)[1] at the Max Planck Institute of Quantum Optics (MPQ), that generates a beam of 21 fs near-infrared (NIR) pulses with a stabilized carrier-envelope phase (CEP) at a repetition rate of either 500 Hz or 1 kHz. For spectral broadening the beam is focused (f = 175 cm) into a hollow-core fiber (inner radius: 275 µm, length: 100 cm) filled with 2.8 bar Ne gas. Consecutively, the octave-spanning laser pulses centered at 720 nm are compressed by a set of chirped mirrors to about 4.5 fs.

Attosecond experiments are performed using the attosecond beamline AS-5 at MPQ shown in Fig. S1(c). The ultrashort, linearly polarized NIR-pulses enter the beamline through a Brewster-angle window. A broadband Ag-mirror with 50 cm focal length focuses the few-cycle laser pulses into a 2.5 mm thick tube filled with Ne gas at a backing pressure of 200 mbar. With an approximate focal diameter of 150 µm, intensities of more than 10^{14} Wcm^{-2} are reached, facilitating high harmonic generation (HHG). The HHG process produces attosecond pulses in the extreme ultraviolet (XUV)[2]. In the following characterization stage of the beamline, a translatable Au-coated mirror is used to deflect the beams either onto an XUV sensitive spectrometer or a beam viewer. In both cases an 800 nm thick Zr-foil attenuates low-order harmonic radiation and the intense NIR beam efficiently. For spectral characterization of the XUV pulse we use a home-built spectrometer with flat-field-corrected grazing-incidence grating with 1200 lines per mm dispersing the beam onto an MCP/phosphor stack. The XUV spectra are recorded by a CCD camera. To image the spatial profile of the XUV beam, the grating is bypassed and the beam directly sent to the MCP/phosphor stack. Deviations from a circular symmetric profile are corrected by minimizing aberrations of the NIR focus in the HHG chamber.

The co-propagating NIR-pump- and XUV-probe-pulses are spatially separated by a 150 nm thick Zr-foil mounted onto a 15 µm thick pellicle fabricated from nitrocellulose. The Zr-foil, situated in the center of the pellicle, is transparent for XUV radiation (with about 60% transmission for 95 eV photons), but blocks the NIR light. Due to the higher divergence of the NIR beam, the NIR beam size is much larger than the XUV beam and part of it is transmitted outside of the Zr foil through the pellicle. In order to avoid damage of the nanotargets, typically occurring at intensities around 10^{12}-10^{13} Wcm^{-2} (Ref. 3), we use coated pellicle filters with reduced transmission of 20% for the NIR. Sensitive adjustment of the NIR intensity used in the streaking experiments is possible with an inline motorized iris.

In the experimental chamber (base pressure: 10^{-8} mbar) the NIR and XUV beams are reflected by a double-mirror setup of 12.5 cm focal length onto the target. The double mirror stack consists of an inner mirror with optimized reflection for XUV light that is surrounded by an outer mirror reflecting the NIR beam. The reflectivity of the inner mirror is centered at 95 eV with a width of 7 eV supporting a maximum reflectivity of 30%. Taking the Zr-foil into account, the total XUV intensity in the spectral region of interest is reduced to about 18% (see Supplementary Fig. 1(a)). Both mirrors in the double stack can be tilted and moved independently in all three dimensions by motorized stages to optimize alignment for spatio-temporal overlap of the two beams. The temporal overlap and the delay between NIR and XUV pulses are adjusted by moving the inner mirror with respect to the outer mirror using a position-encoding high-resolution piezo-stage supporting a temporal resolution better than 10 as. The targets in the experimental chamber can either be a stream of Ne gas emitted from a nozzle or a tapered gold nanowire both mounted on motorized 3D-stages. After impinging on the

target the NIR-pump beam is refocused by a 10 cm focal lens, resulting in the NIR focus being imaged onto a CCD camera outside of the chamber. The recorded image of the focus is used for optimizing the spatial and temporal overlap of the two beams and serves as a tool for coarse positioning of the targets.

The electrons that are generated by the XUV pulse, are detected with a time-of-flight spectrometer (TOF) equipped with an electrostatic lens to enhance detection efficiency for an arbitrary energy regime. In the spectrometer the photoelectrons move along a 70 cm long, field-free drift tube before being post-accelerated onto an MCP detector and recorded via a synchronized multi-scaler card.

Streaking measurements on nanotaper targets were accompanied by recording reference streaking spectrograms for Ne gas. The gas streaking spectrograms were utilized in an iterative procedure to optimize pulse durations and chirp of both NIR and XUV pulses. Furthermore, under the assumption of an instantaneous XUV photoemission, the reference measurement permits to extract the vector potential of the NIR-pump pulse[4] and using an iterative FROG-CRAB algorithm (ATTOGRAM)[5], retrieve the XUV pulse properties. From the Ne gas streaking measurement, shown in Supplementary Fig. 1(b), we obtain an XUV pulse duration of 220 as with insignificant chirp (Supplementary Figs. 2(a) and (b)). The overall count rate in the gas streaking measurement was also used as an indication for the coarse alignment of inner and outer mirror focus position, target position and focus of TOF-extraction optics that should all overlap spatially.

NIR intensities applied to the Au nanotaper never exceeded 1×10^{12} Wcm^{-2} to prevent any damage to the sample observed at higher intensities. Laser foci, target and TOF can be accurately aligned for the tapered nanowire with laser-induced XUV-NIR photoemission. Field enhancement at the apex of the taper supports strong-field emission of photoelectrons up to cutoff-energies of about 15 eV for the used intensities. The high nonlinearity of strong-field photoemission at the taper apex is a crucial prerequisite for the positioning of the nanotaper with nanometer accuracy within the focal plane. The electron count-rate for streaking on the tapered nanowire with about 0.04 to 0.1 counts per shot requires long data acquisition times which are limited by CEP-stability. Streaking data from nanotapers were recorded using delay steps of 100 as with 4000 samples per delay step, leading to an acquisition time for a delay scan of roughly 7 minutes. Depending on the time CEP-stability could be maintained, several delay scans were acquired for an identical parameter set and subsequently superimposed (improving the signal-to-noise in the measurements). In order to exclude long term-drifts, we recorded reference streaking spectrograms with the same settings at regular intervals. The data presented in the main text have been recorded for typically more than three hours for a single streaking spectrogram from the tapered nanowires.

Supplementary Note 2

Sample preparation

Tapered gold nanowires were electrochemically etched in a lamella-drop-off technique from 0.1 mm thick polycrystalline Au wire (99.95 %, Alfa Aesar) using 90 % saturated KCl (Alfa Aesar) in deionized water (in-house)[6]. The gold wire was mounted vertically approximately in the center of two platinum rings (99.95 %, Alfa Aesar), which served as counter-electrode. A brief immersion of the platinum rings in the etching solution forms two lamellas. Etching was performed by applying a potential of +10 V to the gold wire with respect to the platinum rings. Due to formation of crystalline KCl in the upper ring during etching and subsequent rupture the lamella was recovered by reapplying the etchant a few times during etching of one taper. After etching was complete, nanotapers were rinsed with distilled water and acetone to remove remnants of the etching process and inspected with an optical microscope for a smooth and shiny surface (Fig. S6(a)). Nanotapers with radii of approximately 100 nm (Fig. S6(b)) and typical opening angles around 10° ± 4° were obtained.

Supplementary Note 3

Analysis of the streaking spectrograms

After applying a filter to the measured spectrogram, which averages over neighboring data points in order to reduce statistical noise, the cut-off part of the electron kinetic energy spectrum for a single time delay was fitted to a Fermi function

$$f(E_{\mathrm{kin}}) = \frac{A}{\exp\left(\frac{E_{\mathrm{kin}} - E_0}{\Delta E}\right) + 1}, \quad (1)$$

where A, E_0 and ΔE are fitting parameters (see Supplementary Fig. 4(a)). The turning point of the Fermi function, which we use to describe the evolution of the cutoff of the electron spectra, is defined as E_0. The algorithm first finds the energy E' where the edge of the spectrum crosses half of the maximum counts. Then the Fermi-fit is performed in the interval $[E' - 6\ \mathrm{eV}, E' + 20\ \mathrm{eV}]$. The fit is insensitive to the upper bound due to negligible noise and decay of the spectra to zero. For the measurements on the tapered nanowire a plateau-like structure is formed and thus also the lower bound is not critical for the determination of E_0. In the gas streaking measurements by contrast, the XUV-electron spectrum has a Gaussian shape. This leads to a slight dependence of E_0 on the choice of the lower bound of the fitting interval, which, however, merely shifts the streaking curve up or down in energy without affecting the timing. The broad shoulder spanning into the low-energy region (< 75 eV) indicates the presence of a partial coverage of the surface of the nanotaper with higher work function[7]. To minimize the influence of such ad-layers on the results of ANNS, all streaking curves are retrieved by analyzing just the edge of the spectrum. Supplementary Figure 4(b) compares the extraction of streaking curves from a reference streaking spectrogram recorded for Ne using the Fermi-fit (dark blue line) with other methods. In the center-of-gravity method (COG: green line), the center-of-gravity for the spectrum is calculated. The Gauss-method analyzes the peak position of a Gaussian function fit to the gas spectrum (red line). Finally a FROG-CRAB retrieval algorithm is employed[5], which allows both the retrieval of the vector potential and the determination of XUV-pulse properties. There is an overall good agreement between all methods. A slight variation in the amplitude is visible but the timing agrees almost perfectly, especially between Fermi-fit and FROG-CRAB retrieval. The good agreement of all methods is also related to the quasi chirp-free attosecond XUV-pulses in our experiments (see Supplementary Fig. 2) thanks to excellent chirp compensation of the focusing multilayer mirror.

The extracted streaking curves are smoothed by a Fourier-filtering algorithm. First, the extracted streaking curves are zero padded to obtain a better spectral resolution and then Fourier-transformed via numerical FFT. A filter is applied to the resulting spectra, which rejects all the low-wavelength components below 400 nm and possesses a linear transition up to 500 nm, above which all components are accepted. The spectral cutoff of the experimentally employed NIR-pulses usually lies at the upper edge of this wavelength region. Additionally the part of the FFT-spectrum which corresponds to negative frequencies is set to zero, and the positive frequency components are consequently multiplied by a factor 2. Upon inverse Fourier-transform a smoothed complex valued curve is obtained, whose real part describes the streaking curve and which allows through the complex values the extraction of the envelope and the phase of the streaking curve. This approach facilitates the straightforward calculation of the derivative of the streaking curve, needed for the reconstruction of the electric field.

Supplementary Note 4

Electric field and response function retrieval

In the ponderomotive streaking regime (discussion see Chapter 2.7) the final change of momentum $\Delta\mathbf{p}$ of the electrons emitted at time t_0 can directly be related to the component of the vector potential $\mathbf{A}$ parallel to the emission direction by solving the classical equation of motion (in the Coulomb gauge): $\Delta\mathbf{p} = -e\,\mathbf{A}(t_0)$. Expressing this equation through energies and taking the derivative, allows a direct reconstruction of the local electric field E from the energy shift ΔE_{kin} of the kinetic energy of the electrons measured in the streaking spectrogram:

$$E(t_0) = \frac{1}{2e}\frac{1}{\sqrt{2m_{\mathrm{e}}(E_0+\Delta E_{\mathrm{kin}})}}\frac{\partial\Delta E_{\mathrm{kin}}(t_0)}{\partial t_0}\ , \tag{2}$$

where e is the electric charge, m_{e} the mass of the electron and E_0 is the initial kinetic energy. ΔE_{kin} and E_0 are obtained from the extracted streaking curve. Since the complex valued smoothed curves are directly employed in the calculation, both amplitude and phase of the electric field can be obtained.

In the framework of linear Maxwell's equations, external excitations are discussed in terms of response functions in the frequency domain. For a fixed geometry of particle and source, the electric field vector at position *r* and frequency ω can be written as

$$\mathbf{E}(\mathbf{r},\omega) = \mathbf{H}(\mathbf{r},\omega)\cdot\mathbf{E}_{\mathrm{input}}(\omega), \tag{3}$$

Where $\mathbf{E}_{\mathrm{input}}(\omega)$ is the electric field vector of the source and $\mathbf{H}(\mathbf{r},\omega)$ is the response of the system at position $\mathbf{r}$ to the given excitation, which is a 3 × 3-matrix. Since the streaking in our experiment focuses on the electric field component parallel to the axis of the tapered nanowire and an input beam with the same polarization, one component of the above response-matrix is sufficient and the above relation can be understood in a scalar sense. The response is separated into the absolute value $abs(\mathbf{H})$ and the phase $\varphi(\mathbf{H})$, such that $H = abs(\mathbf{H})\cdot e^{i\varphi(\mathbf{H})}$. We use the convention in which the response function for the nanotaper is normalized by the free space response, i.e. the phase shift due to the propagation of the incoming pulse is eliminated, as is measured by attosecond streaking. In our experiment the streaking trace is composed of electrons originating from different emission points. With the straight-forward analysis described above the retrieved electric field from the nanotaper will thus be averaged over the area subjected to the XUV-pulse. The variation of the near field around the tapered nanowire is, however, small, which is the reason for the obtained homogeneous streaking traces (see also Supplementary Note 8). Due to the limited statistics of our experiments, the apex contribution is not visible. The response function is retrieved by comparing the gas to the nanotarget streaking measurements as $H = E_{\mathrm{nanotip}}/E_{\mathrm{gas}}$. Since the overall covered delay range and the delay steps are generally not identical between the two different measurements, zero-padding of the delay scans has been applied to increase the resolution in the Fourier transform. Then linear interpolation has been applied to compute the response at the frequencies given by the original (not zero-padded) gas measurement (crosses in Fig. 4 (b) and (c)). The averaged response is computed from the zero-padded Fourier transforms and weighted by the statistics of the individual measurements. Since the measured and theoretical response are almost wavelength-independent in our experimental geometry, the relative phase and amplitude can be directly seen when comparing

the reconstructed electric fields around the nanotaper in the time domain (see Supplementary Fig. 5).

Remarkably, despite the relatively high incoming intensities, the measured electric fields agree well with the fields calculated using linear macroscopic Maxwell's equations (see Fig. 4c). Details of the calculation of the expected response function can be found in the Supplementary Note 5. The data points show a slightly higher spread than expected from theory. The reason for this might be that the gas streaking probes the entire focal volume while the nanotarget only samples a small portion of the laser focus, which makes the measurements more sensitive to spatial inhomogeneities. The averaged response function for wavelengths longer than 850 nm seems to show a slight systematic deviation from the expectation. Whether this might be solely due to small statistics or is due to nonlinear or quantum effects that are not accounted for in our field calculation using Maxwell's equation is not clear.

As has been observed under comparable conditions before[8], the laser-field peak intensity in the focus can be significantly higher than expected from the streaking amplitude recorded in a gas streaking measurement. Using gas targets, electrons originate from the entire XUV focal volume, effectively integrating over a range of NIR intensities. In contrast, the nanotaper can be precisely placed in the NIR focus, using our procedure shown in Figure 2, and focal volume integration can be neglected. We correct for the underestimated field amplitude in the gas streaking measurements by comparing them with the cutoff energies of the direct strong-field electron emission (for every measurement) from the enhanced apex near-fields. Correction factors of around 3 are obtained in agreement with results from earlier experiments on gas streaking at higher intensities[7]. This also solved the discrepancy between the observed cutoff of the NIR emitted electrons from the tapered nanowire and the input intensity, just estimated from the gas streaking, which would have required field enhancement factors of around 12, which is unrealistically high for the apex radii of the nanotapers used in our experiments.

In our proof-of-principle experiments the resolution of the individual measurements is limited due to the rather short covered delay times. In future measurements, especially if the response contains sharp resonances, longer delay ranges need to be covered. For the reconstruction of the electric field from different spots, e.g. apex contribution vs. side of the tapered nanowire, more sophisticated algorithms for the analysis of the streaking curves are necessary. We believe that by increasing the XUV flux and reducing the spotsize, the measurements can be improved in the future.

Supplementary Note 5

Details of the simulations

For the simulation of the attosecond streaking traces a classical Monte-Carlo algorithm was used. The electromagnetic fields induced by the NIR-pulse around the metallic nanotaper are calculated using a finite-difference-time-domain (FDTD) solver of Maxwell's equations (FDTD Solutions, Lumerical)[9]. The tapered nanowire is modeled as a semi-infinite cylinder of radius 100 nm with a semi-sphere as apex. The refractive index of Au is fitted to the data of Johnson and Christy[10]. The intensity full-width-at-half-maximum (FWHM) of the Gaussian NIR input beam is 15 μm. The temporal profile is modeled as Gaussian with center wavelength of 720 nm and intensity FWHM (T_{FWHM}) of 4.5 fs (used in the following unless otherwise stated):

$$E(t) = \exp\left(-2\ln 2 \cdot \left(\frac{t}{T_{\mathrm{FWHM}}}\right)^2\right) \cdot \cos(\omega_0 t + \varphi_{\mathrm{CE}}), \tag{4}$$

where ω_0 is the center frequency and φ_{CE} is the carrier-envelope phase (CEP). For the calculations of the electric fields, a CEP of 0 was used. The simulation box was 20 μm × 20 μm × 1.6 μm using absorbing boundary conditions, and a grid step size at the apex of 2 nm.

The XUV beam is modeled as a Gaussian in space with a spatial intensity FWHM of 5 μm and temporal intensity FWHM of 220 as, the value retrieved from the Ne streaking measurements (Supplementary Fig. 2). We treat the XUV beam in ray approximation. Randomly chosen points in the Gaussian beam profile are propagated in the direction of the beam. For rays hitting the Au nanotarget an electron is released at its surface at a randomly chosen delay within the delay times of interest. From all angles, under which electrons can be released, we select those electrons with their final direction within the detection cone of the experimental TOF-spectrometer with an opening angle of 45°. Note that the streaking field for the intensities used in our experiments does not significantly change the direction. The initial kinetic energy is given by the experimentally measured TOF-spectrum from the nanotaper, as has been done in Ref. [11]. The emitted electrons are propagated in a fully three-dimensional simulation, employing the classical equation of motion:

$$\mathbf{v}(t) = -e\int_{t_0}^{t} \mathbf{E}_{\mathrm{NIR}}(\mathbf{r}(t'), t')dt' + v_0, \tag{5}$$

where $\mathbf{E}_{\mathrm{NIR}}$ is the full three-dimensional electric field due to the NIR pulse, $\mathbf{r}(t)$ the position of the electron at time t and t_0 is the emission time. This relation neglects the magnetic field component, which is a reasonable assumption for the considered electron energies and laser intensities. The propagation is conducted numerically with the help of the fields calculated above using a Velocity-Verlet algorithm[12]. The employed time-steps have been checked to give converged results. The single energy streaking curves (Fig. 3(d)) have been calculated using the same basic algorithm with a fixed initial energy of 89 eV and an emission angle of 15° with respect to the surface from specific emission points at each delay step. The angle is chosen to be roughly the mean emission angle of all electrons detected in the TOF spectrometer. The reference streaking curves were calculated directly from the vector potential of the incident laser pulse (see methods and eq. 10, Supplementary Note 9, below). Supplementary Figure 6 compares an experimentally measured streaking trace with the MC simulation. Good agreement between both traces can be observed. In the simulation a comparison between the reference streaking trace and the contribution from the tapered nanowire yields a delay

of Δt=-300 as and a relative amplitude of about 0.5, that is expected from the electric near-field (see Figure 1 (d)), which is close to the mean value of the measurements (see Fig. 4).

For the calculation of the expected response function a conical tip with full opening angle of 10° and a hemi-sphere of radius 50nm at the end has been assumed. The laser beam was modeled as a Gaussian beam with 15 μm, as above, however with a pulse form spanning a wavelength range from 400-1200nm. The local response function as defined above was contained by Fourier-transforming the electric field in polarization direction at the surface and dividing it by the Fourier-transform of the incoming pulse, i.e. the pulse in the focal point without tip. The response is normalized to the free-space response as discussed above. To obtain the expected response function, the response of 10 000 points on the surface, weighted by the XUV-profile, has been averaged. The width of the expectation in Fig. 4 b)-c) is given by the standard deviation. The response function shown in Fig. 1(d) in the main text is calculated using a Gaussian NIR beam, focused onto the tapered nanowire, with a spatial intensity FWHM of 15 μm. For the results calculated using Mie theory, the input beam is a plane wave with the polarization parallel to the cylinder axis.

The reason that a conical has not been assumed in the streaking simulations is given by the occurrence of artificial hotspots at the surface from rectangular gridding of the simulation volume in FDTD, which has also been observed in Ref. [13]. This limitation can be overcome by using a significantly finer mesh, however at the cost of massively increased memory requirements, which hinder the subsequent calculation of electron propagation with our available computational resources. For the conclusions of the simulations this is however irrelevant as the fields and response functions can fully be understood in the framework of infinite cylinders as is discussed in Supplementary Note 6.

Supplementary Note 6

Near-fields for different nanotaper geometries

In Supplementary Fig. 7 the influence of the nanostructure geometry on the near-fields is shown. Supplementary Fig. 7(a) schematically shows the geometry. The point at the surface facing the incoming beam has been chosen as a representative point. The component parallel to the cylinder axis, in detection direction, is investigated. The model for the tapered nanowire as a semi-infinite cylinder as used in the streaking simulation is an idealization, since the nanotapers used in our experiments have a conical shape with opening angles around 10°-20° (see Supplementary Fig. 3). Supplementary Fig. 7(b) compares the electric fields for a nanotaper with opening angle of 12.5° and an apex radius of 50 nm (blue line) and a semi-infinite cylinder (green line) at a distance of about 3 μm from the apex to an infinite cylinder (red line). The radius of the cylinder is 350 nm, which, for the nanotaper, corresponds to the distance of the investigated point to the cone-axis. All three lines show excellent agreement in the main pulse ($t < 7.5$ fs). For the nanotaper and the semi-infinite cylinder a second pulse with considerably smaller amplitude arrives at later times. It can be identified as a traveling plasmon excited at the apex, which is absent for the infinite cylinder. The arrival time of the surface plasmon depends on the distance from the apex. For decreasing distances the surface plasmon overlaps with the main pulse and leads to a small distortion. Since in our experiment a region that is considerably bigger than the wavelength of the plasmon is probed, these small distortions are, however, averaged out, leading merely to a slight broadening of the streaking trace and the averaged near-field response. Altogether, it can be concluded that also for tapers with small opening angles, the infinite cylinder model is a good approximation for investigating the response of a tapered nanowire. This has also been used in the theoretical investigation of adiabatic nanofocusing of surface plasmons.[14] This significantly simplifies the treatment, as an analytic solution in form of Mie-theory is known. Nevertheless, in the simulation of the streaking traces the nanotaper was modeled as a semi-infinite cylinder in order to investigate the potential contribution of electrons emitted from the apex.

We use the response function to discuss the effect of nanotaper geometry variations on the electric field. The response function can easily be obtained from numerical calculations of the fields in the frequency domain, but also from time-domain calculations using Fourier-transform.

The finite opening angle of nanotapers leads to an increase of the radius with the distance from the apex. Supplementary Figure 7(c) shows the response function in the infinite cylinder approximation for different radii, 50 nm (light blue), 100 nm (blue), 350 nm (green) and ∞ (i.e. a flat surface, black dashed line) at the same position as in (a). The absolute value $\mathrm{abs}(\mathbf{H})$ (left panel) exhibits a moderate decrease towards higher wavelengths and changes only slightly with increasing radii. The phase (right panel) shows a flat behavior in the region relevant for the pulses used in the experiment (500 nm-1100 nm), but is shifted with increasing radii towards more negative phases, which implies an increase in the shift between the streaking from the tapered nanowire and gas streaking. Considering nanotapers with radii between 50 nm and 3000 nm and an increase of the radius over the XUV-focus due to the finite opening angle of around 10°, the expected phase shift for our experiment lies between -0.7 and -1.1 rad. The spread is still small compared to the optical period of 2π. This is close to the result obtained by averaging the response over different emission points. The change of the response function around 500 nm is connected to a change of the refractive index stemming from intraband transitions from the d-band in gold. For increasing radii the response

asymptotically approaches the response of a flat surface. As discussed above, the propagating plasmon additionally slightly broadens the response function.

Streaking from different positions on the nanotaper

So far the discussion of the response of the nanotaper has been confined to points facing the incoming beam. Electrons will, however, also be emitted from other points at the front side of the cylinder. Electron emission from the backside of the nanotaper (i.e. θ>90°) is negligible due to the strong absorption of XUV photons in Au. Using the result of Fig. 3(d) and the discussion in the next section, i.e. the ponderomotive streaking regime for our parameters, the vector potential can be used to obtain the final streaking curve. Supplementary Figures 8(a) and (b) show the temporal shift and the amplitude of the vector potential around the cylinder (white circle) relative to the input vector potential, for a radius of 100 nm and an input pulse of 4.5 fs FWHM and center wavelength of 720 nm. As can be seen, the shift of the vector potential is approximately constant at the front side of the cylinder, while the amplitude slightly decreases with increasing angle θ. It can also be noticed that the change of the vector potential in space takes place on the length scale of the radius of the nanotarget. Emitted electrons are only born close to the surface due to the small mean free path for inelastic scattering**.** Supplementary Figures 8(c) and (d) show the shift and the relative amplitude around the surface for different radii. The shift is practically constant for all angles around the cylinder and shifts with increasing radius similar to the electric fields. The amplitude shows a decrease with the angle. Since the electrons emitted from different angles around the nanotarget are, however, in phase, a homogeneous streaking trace can be expected even for radii > 1 μm. Together with the discussion of Supplementary Fig. 7, it can be concluded that the expected qualitative results do not depend very sensitively on the radius. It should be noted, that the field experienced by the electrons varies slightly in amplitude and phase. This means that in our experiments the electric field reconstructed from the retrieved streaking curve, can only be regarded as an average field. Especially for electrons emitted from the side of the tapered nanowire, the variations are, however, relatively small. The reconstructed field therefore describes the actual electric field at the nanotaper shank very well. This is different for other geometries studied in the literature, e.g. plasmonic nanospheres,[15] where different surface electric fields, which vary significantly in amplitude and phase, contribute to the streaking spectrogram. In that case a more sophisticated retrieval algorithm has to be applied to reconstruct the different contributions.

Influence of adsorbates on the measured phase shift

The limited vacuum conditions in our experiment cannot guarantee a perfectly clean surface of the gold nanotaper and the formation of a thin overlayer by adsorption of particles from the background gas is expected. Supplementary Figure 9 shows the influence of a 5 nm thick overlayer on the electric field at the gold nanotaper for arbitrary refractive indices, calculated using a wavelength of 720 nm and a radius of 150 nm. Adsorbate materials in this wavelength region will have a refractive index with $Re(n)<2.5$ and $Im(n)<5$, which gives even for layers with a few nm thickness an additional shift below 100 as.

Supplementary Note 7

Visibility of the apex contribution

In our current experiments we were not able to clearly detect a streaking signal from the nanotaper apex. As indicated by numerical simulations, this is due the limited statistics. A simple estimate for the number of electrons n_P from a certain region P (e.g. the apex), neglecting details of the photoemission process, yields:

$$n_P \propto \frac{n_{\mathrm{XUV}}}{d_{\mathrm{foc}}^2} \cdot A_P \cdot f_{\mathrm{rep}} \cdot T_{\mathrm{acq}}, \tag{6}$$

where n_{XUV} is the number of XUV-photons in the incoming beam, d_{foc} is the diameter of the XUV focus, A_P is the cross sectional area of the region of interest in beam propagation direction, f_{rep} is the repetition rate and T_{acq} is the overall acquisition time. By comparing our experiments with numerical simulations, where the apex contribution is clearly visible, see Supplementary Fig. 10, an estimated improvement of the counts from the apex by roughly three orders of magnitude is necessary in order to detect the respective streaking signal. From Eq. (6), several parameters for such an improvement can be identified. First, tighter focusing of the XUV beam will help to increase to signal, however, the focal spotsize and useful focal length are limited by the realizable surface quality of the focusing mirrors and geometrical restrictions in the experimental setup. We estimate that this limits such an improvement to about a factor of $\sim 50 - 100$. Although the other parameters are much harder to improve and the acquisition time of a few hours for a single scan is already close to the limit of what seems possible at the moment, using higher repetition rate attosecond sources (providing a factor $3 - 10$), and lower XUV-photon energies to increase the flux while still being separated from the NIR background (factor ~ 5), we believe that characterization of the electric fields of features even as small as the nanotaper apex seem to be possible in the future. We additionally note that in order to detect the signal from the apex over the strong background of the taper contribution, a certain minimum streaking amplitude is necessary. The cutoff of the XUV-photoemission spectra is not sharp but has a finite width ($\sim 3 - 4$ eV), due to the width of the density of states and the shape of the XUV-spectrum. Electrons from the apex, with final energies in the cutoff region or below will not be distinguishable from the taper signal (see Supplementary Fig. 10). Neglecting the relative phase of the two contributions, considering relative delays <800 as, we conclude that the streaking amplitude of the apex signal should be higher than that from the taper by at least the width of the cutoff region. With the used intensities we lie slightly above that threshold but with higher intensities the visibility could be improved, where the maximum intensity is limited by the cutoff of the NIR background and potential damage of the nanotarget.

Supplementary Note 8

Finite photoemission delays

In the above model and analysis it has been assumed that directly after the excitation the electrons follow a free electron motion in the external laser field. This is the well-known strong-field approximation (SFA) of strong-field physics[16]. Under this approximation the equation of motion reads:

$$\mathbf{p}_{\mathrm{f}}(\tau) = \mathbf{p}_0 - \int_{\tau}^{\infty} \mathbf{E}(t)dt = \mathbf{p}_0 - \mathbf{A}(\tau), \tag{7}$$

where $\mathbf{p}_{\mathrm{f}}$ is the final momentum of an electron released at time τ by the XUV-pulse, $\mathbf{p}_0$ is the initial momentum and $\mathbf{E}(t)$ and $\mathbf{A}(t)$ the electric field and vector potential of the external laser. The electron charge e has been set to 1. This relation permits the extraction of the vector potential and reconstruction of the electric field of the laser pulse. In reality, however, the above assumption is not strictly valid and several effects can result in a slight modification of the above relation, which may be interpreted as a non-instantaneous photoemission in terms of a streaking delay τ_{S}. This is the effective delay between excitation of the electron by the XUV pulse and emission into the streaking field. The modified relation is usually expressed as[17]:

$$\mathbf{p}_{\mathrm{f}}(\tau) = \mathbf{p}_0 - \beta \cdot \mathbf{A}(\tau + \tau_{\mathrm{S}}), \tag{8}$$

where β is an additional factor, which takes the modification of the streaking amplitude into account. In the following, we will focus on the discussion of τ_{S}. We will shortly introduce the different effects which contribute to the streaking delay and argue why they can be neglected in our experiment. For a review and introduction to streaking delays, we refer to Refs. [18-20].

Directly after ionization the released electron feels the potential of the remaining ion. The photoemitted electron will therefore arrive at the detector at a different time compared to a free electron, which starts at the same position and time with identical (final) kinetic energy. The average arrival time also depends on the starting point and therefore on the electron distribution of the initial and final state. The difference in the arrival times between a free electron and an initially bound electron has been termed Eisenbud-Wigner-Smith-delay τ_{EWS}. While attosecond streaking experiments which measured the relative delay time in neon[21] were initially discussed in terms of τ_{EWS}, it soon turned out that the presence of the strong NIR streaking field itself leads to additional contributions to the measured delays. For attosecond streaking from atoms the streaking delay can be decomposed into different contributions[20]:

$$\tau_{\mathrm{S}} = \tau_{\mathrm{EWS}} + \tau_{\mathrm{CLC}} + \tau_{\mathrm{dLC}}^{i} + \tau_{\mathrm{dLC}}^{f}, \tag{9}$$

Where τ_{CLC} is the Coulomb-laser-coupling-delay, which occurs due to the change of the electron momentum under the influence of the Coulomb field of the remaining ion (Coulomb-Laser-Coupling).[16,22,23] τ_{dLC}^{i} and τ_{dLC}^{f} are the delays caused by the coupling of the laser field with the dipole moment of the initial and final state, respectively.[20,24] Interestingly, τ_{S} can take on positive and negative values. The streaking delay is closely related to delays found in other laser-assisted photoemission schemes, e.g. using the RABBIT technique[25]. On solid surfaces the description of streaking delays is more complex. Here, screening of the laser field inside the material, propagation

of the electron through the crystal lattice, and interaction with the other electrons and the image charge have to be accounted for.

While it is not possible to measure an absolute streaking delay, several experimental studies focused on measurements of the relative streaking delays between two or more initial states in gases[21] or on plane metal surfaces[26-29]. Relative delays for photon energies around 100 eV are on the order of 20 as for noble gases and can reach up to 100 as for surfaces. For photon energies around 35 eV delays of 100 as have been measured in Argon using RABBIT.[30,31] So far, experimental studies that directly compare the relative streaking delays from a gas to a solid are lacking. In order to estimate the importance of streaking delays on the outcome of our simulations and the electric field reconstruction, a comparison of absolute streaking time delays, which are only accessible through theory, is necessary.

The first reported experiment for neon[21] was followed by a number of theoretical publications which aimed to explain the measured time shift with different theoretical methods[32-36]. The absolute time delays for photoemission from the 2p-state, discussed either in terms of τ_{S} or τ_{EWS}, were found to lie below 10 as for photon energies around 100 eV. Such a delay is hardly detectable in our setup and can therefore be safely neglected for the measured shift between the streaking traces of the tapered nanowire and neon.

For the photoemission from solids the comparison is more complicated. The pioneering experiment recording relative delays between core and valence bands for a plane tungsten surface[28] triggered a considerable amount of theoretical work.[37-44] While in our experiment electron emission should locally be describable as occurring from a flat surface, previous work cannot be strictly compared to our experiment. So far, practically all theoretical studies focused on streaking delays, where the electron emission and streaking field polarization are normal to the metal surface. In this case the electromagnetic field is screened inside the solid on a length scale even shorter than one atomic layer,[27] while the XUV penetrates a few nanometers into the material. As a consequence, although inelastic scattering limits the emission depth to the order of 1 nm, electrons born inside the material need some time before they reach the surface and only then experience the streaking field. This mechanism has been highlighted in a recent study.[27] For such a geometry quantum mechanical simulations carried out within the single active electron approximation suggest that the absolute photoemission delay from the valence band of a metal for a photon energy of 100 eV will be below 100 as.[38,39,42] In our experiment, for the electrons emitted from the nanotaper, the situation is different and detected electrons are emitted quasi parallel to the surface. They therefore effectively probe the laser component parallel to the surface. According to Maxwell equations this component is continuous across the surface and quasi homogeneous over the electron emission depth. The electrons are therefore already subjected to the full streaking field, even when they are still inside the solid. Additionally considering the polycristallinity of the probed surface and relatively high kinetic energies of around 100 eV, free-electron like dispersion can be assumed. Due to the averaging over all different crystal orientations, effects related to resonances and transitions close to band gaps[42,44] should not play a dominant role. That means that free-electron like propagation of the electron in the external field on its way to the surface, is a good approximation. Hence, in contrast to conventional attosecond streaking schemes on plane surfaces, the delay due to the electron propagation to the surface should be negligible.

The interaction of the excited photoelectron with the remaining hole is strongly screened inside metals and for valence band electrons usually neglected in the theoretical studies. For screened atomic potentials theoretical studies have employed Yukawa-potentials, which can also be used in the description of screened Coulomb interactions inside solids. These studies suggest that the streaking delay ($\tau_{\mathrm{EWS}} + \tau_{\mathrm{CLC}}$) for screening length on the Angstrom length scale, as typically found in metals, are on the single attosecond time scale, especially if emission occurs from a delocalized state.

An examination of the streaking delay due to dynamic image charge interaction in the conventional surface streaking geometry is given in Ref. [38]. The image charge interaction leads to a Coulomb like potential along the surface normal on the vacuum side. As the Coulomb-laser-coupling can reach up to few 100 as for low-energy electrons[23] and the electrons detected from the side of the nanotaper possess only little kinetic energy along the surface normal, one might expect a considerable impact of Coulomb-laser-coupling. The streaking field and Coulomb-like force are, however, perpendicular and therefore decouple. This can best be understood in the quasi-classical treatment of τ_{CLC}.[20] A streaking delay due to CLC with the image charge interaction potential can therefore safely be neglected.

As the name suggests, the dipole-laser-coupling streaking delay τ_{dLC} in atoms is caused by a coupling of the laser streaking field with a induced or permanent dipole moment of the atom and there is an ongoing debate whether a change of the dipole moment during the emission is necessary for this contribution to come into effect[20,24]. What is overlooked in this discussion is that besides leading to a coupling of different initial or final states, the dipole coupling to the laser field will lead to an additional dipolar electric field contribution around the atom, which is generally out of phase with the driving laser. Therefore even when the dipole moment does not change during emission, there is generally a delay in the streaking trace due to the different electric field, compared to the case in which a dipole coupling is absent. In the case of solids the emitted electron density due to the XUV-field and strong streaking field at intensities used in our experiments is negligible compared to the electron density in the valence band. Therefore the response of the solid is effectively not altered during the emission. However, the dielectric response of the solid leads to a change of the electric field compared to the incoming laser field, comparable to the dipole response of a single atom. In our case the response can be traced back to the collective free-electron-like polarization response of gold in the wavelength region of the exciting laser (see e.g. Ref[45]), but it still depends on the geometry. The response is in principle dependent on the wavelength of the exciting laser, and since it might change within the covered bandwidth, the simple equation (8) using a single streaking delay does not suffice for the description anymore. The observations of our experiment are therefore not described in terms of a single streaking time shift and amplitude, but in terms of a change of the electric near-field, compared to the electric field of the incoming laser pulse. Using the terms of the response function, the frequency dependence of this response can be reconstructed, which would not be possible using equation (8). Nevertheless, the effect measured in this work can be regarded as a many-body-analogue of the dipole-laser-coupling in atomic streaking.

Electron interaction effects were discussed in a number of publications[32,36,46] to explain the slight discrepancy between experiment and theory in attosecond streaking from neon. One of the effects which was identified as a possible source, is the influence of shake-up states[36]. Due to electron-electron interactions, at the same time an electron gets emitted, another electron is transferred to an excited state. The photoemitted electron will end up at slightly lower kinetic energies and with an

additional delay. If this electron ends up in the lower part of the spectrum, it might influence the extracted delays when an algorithm is employed, which uses the first moments of the streaked electron spectrum. For neon this effect was found to be on the order of 10 as. In a solid a similar mechanism would be the excitation of bulk or surface plasmons. This effect is probably one of the reasons for the shoulder of our spectrogram towards the lower energy region. Since we, however, only use the curve extracted at the cutoff, our measurements are unaffected by such emission channels.

Finally, since our results can be explained well in terms of the electromagnetic response of the nanotaper, we conclude that the assumption of a negligible streaking delay is reasonable. We note, that only recently an experiment using the RABBITT technique was realized, which was able to extract the difference in emission times between noble gases and noble metal surfaces[47], and found a delay on the order of 100 as for the case of argon and gold for electrons with energies around 30 eV, however with a large contribution of propagation effects and in the conventional emission geometry. As above these delays are expected to be much lower for electron energies around 100 eV and for our geometry.

Effects of the strong NIR fields

In order to assess additional effect of the strong NIR-laser field on the solid and the XUV-emitted electron dynamics at the streaking intensities used here, such as space-charge interaction of electrons and a perturbation of the electron distribution function, it is instructive to examine other attosecond streaking experiments from solid surfaces. Although they use higher NIR-intensities and have a much larger emission area, they do not report the observation of such effects. Concerning space-charge interaction it has to be noted that in our experiment most NIR electrons come from the nanotaper apex, while XUV-electrons, which we use to extract the electric field, come from the tapered nanowire. They are hence spatially separated already at the time of emission. Since the velocity of the XUV electrons is much higher, they pass the apex region when the NIR electrons are still located close to the apex. The electric field of the NIR electrons and the respective image charge will lead in first order to a dipole field, which, however, is negligible at the distance at which XUV-electrons pass the apex compared to the streaking field. For XUV-electrons emitted from the apex, the situation might be different, but since they are not considered in the analysis, this effect is omitted. We also note, based on the count rates, that only a few NIR electrons are emitted per shot. A certain amount of NIR photons will be absorbed in the solid, which leads to a modification of the distribution function close to the Fermi edge. This might be important for strong-field photoemission from solids[48-50]. However for gold the density of states is low at the Fermi edge compared to the (unaffected) d-band, such that the effect can be neglected for the linear XUV photoemission process discussed above.

Supplementary Note 9

Streaking regime

As discussed above, in conventional attosecond streaking from gases or molecules the attosecond streaking trace can directly be related to the vector potential $\mathbf{A}$ of the laser pulse at the time of emission t_0,[31,32]

$$\Delta E(t_0) = -m_\mathrm{e} \cdot \mathbf{v}_0 \cdot e\mathbf{A}(t_0) + \frac{1}{2} m_\mathrm{e} e^2 \mathbf{A}(t_0) \tag{10}$$

where ΔE is the change of the kinetic energy of the electron, $\mathbf{v}_0$ is the initial velocity of the electron, and e is the electron charge. The vector potential is connected to the electric field $\mathbf{E}$ of the laser pulse in the Coulomb gauge via

$$\mathbf{E}(t) = -\frac{\partial \mathbf{A}(t)}{\partial t} \tag{11}$$

The spatial decay of the near-field drastically changes the relation of the fields at the surface to the streaking curve, both in phase and amplitude. While in streaking from atomic systems the streaking curve is related to the vector potential, in attosecond streaking from nanoobjects three different regimes can be identified (Supplementary Fig. 11(a)). The regimes depend on the adiabaticity parameter δ, the ratio of the near-field decay length l_nf to the distance l_{eT} the electron travels within one optical cycle, or equivalently, the time it takes the electron to leave the near-field T_esc to the optical period T_0:

$$\delta = \frac{l_\mathrm{nf}}{l_{eT}} = \frac{l_\mathrm{nf}}{v_0 \cdot T_0} = \frac{T_\mathrm{esc}}{T_0}, \tag{12}$$

where v_0 is the initial velocity of the electron. We define l_nf here as the $1/e$ –decay length

a) Ponderomotive regime, $\delta \gg 1$:

   The near-field decay length is much greater than the distance the electron travels in one optical cycle. The electron therefore effectively experiences a homogenous field. This case is identical to streaking from atomic systems. The streaking curve is directly related to the vector potential at the surface of the nanoobject.

b) Instantaneous regime, $\delta \ll 1$:

   The electron leaves the near-field within a fraction of the optical cycle. It can therefore be thought of as being accelerated by a static electric field[51]. The streaking curve is directly related to the electric field at the surface, therefore also called 'field-probing regime'. The energy changes approximately as $e \cdot l_\mathrm{nf} \cdot E_\mathrm{nf}(t_0)$.

c) Intermediate regime, $\delta{\sim}1$:

   The electron stays long enough in the near-field regime to experience the temporal variation of the field, but it leaves it fast enough, such that the spatial variation of the field influences the streaking curve.

For a field that is exponentially decaying normal to the surface and under the assumption of an exponential decay in time, an analytic solution has been found relating the streaking curve to the electric field at the surface[18,52] by integrating the equation:

$$\Delta\mathbf{p}(t_0\,) = -e_0 \int_{t_0}^{\infty} \mathbf{E}(\mathbf{r}(t), t)dt = \; -e_0 \int_{t_0}^{\infty} \mathbf{E}_0 e^{(i\omega - 1/\tau_\mathrm{p} - v_0/l_\mathrm{nf})\cdot t} e^{+t_0/\tau_\mathrm{p}}\, dt\,, \qquad (13)$$

where τ_p is the temporal decay constant of the near-field, and v has been assumed constant for describing the propagation of the electron, which is a reasonable assumption for typical streaking intensities. The phase shift φ_nf between the electric field at the surface and the streaking curve is given by

$$\varphi_\mathrm{nf}\big(\delta, T_0, \tau_\mathrm{p}\big) = -\pi + \arctan(\frac{2\pi\cdot\delta\cdot\tau_\mathrm{p}/T_0}{\delta + \tau_\mathrm{p}/T_0}) \qquad (14)$$

and the phase shift with respect to the surface vector potential

$$\varphi_A\big(\delta, T_0, \tau_\mathrm{p}\big) = \arctan\big(2\pi\cdot\tau_\mathrm{p}/T_0\big) - \arctan(\frac{2\pi\cdot\delta\cdot\tau_\mathrm{p}/T_0}{\delta + \tau_\mathrm{p}/T_0}). \qquad (15)$$

For the instantaneous regime we obtain $\varphi_\mathrm{nf} = -\pi$ and the streaking curve is proportional to -1 times the electric field (due to the negativity of the electron electric charge), while for the ponderomotive regime $\varphi_\mathrm{A} = 0$. The shift of the streaking curve (φ_A) with respect to the parameter δ is shown in Supplementary Fig. 11(b) for different ratios τ_p/T_0. In the ponderomotive regime, for broadband/ultrafast excitations, the above result doesn't depend on whether the near-field really approaches zero or whether it reaches some other value, since the pulse has already decayed temporally before the electron leaves the near-field region.

In the instantaneous limit for broadband excitations the exciting pulse is still present outside the near-field region and strongly influences the electron dynamics after it leaves the near-field. This is different for the ponderomotive regime, as the exciting pulse will not be present anymore once the electron has left the near-field. Furthermore any post-pulse will not change the energy of the electron. This has been confirmed in simulations of attosecond streaking from nanospheres of different sizes.[15] We note that the above discussion and especially the last point is very similar to the case of strong-field photoemission from the apex of metallic nanotips.[53,54] Therefore, while the above formula might become inaccurate in the instantaneous limit, it is very useful in estimating whether the experiment has been conducted in the ponderomotive regime. As a rule of thumb, the near field decay length is on the order of the geometric features of the nanoobject, in our case the radius of the tapered nanowire. The length scale of the change of the near-field in our experiment is thus on the order of (30 – 100) nm and depending on the angle, the electron velocity away from the surface is about (0-3) nm fs^{-1}, which for an NIR pulse ($T_0 \approx 2.5$ fs) gives a minimum δ of about 3. In contrast to the assumption made in deriving the above equation, close to the surface of the nanotaper the field is not decaying but increasing, as can be seen in Supplementary Fig. 8(b). Allowing negative values for δ in eq. 15, a shift of around -30 as is obtained. The numerically calculated shifts of around -20 as (see Supplementary Fig. 12) are in surprisingly good agreement with the estimation through eq. 15. The obtained shifts clearly show that the experiments are in a ponderomotive regime, which directly allows the reconstruction of the electric near-fields at the surface of the tapered nanowire with negligible error from the measured streaking traces using eq. 2 (see also methods).

Supplementary References

1 Ahmad, I. *et al.* Frontend light source for short-pulse pumped OPCPA system. *Appl. Phys. B* **97**, 529-536 (2009).
2 Krausz, F. & Ivanov, M. Attosecond physics. *Rev. Mod. Phys.* **81**, 163-234 (2009).
3 Summers, A. M. *et al.* Optical damage threshold of Au nanowires in strong femtosecond laser fields. *Opt. Exp.* **22**, 4235-4246 (2014).
4 Goulielmakis, E. *et al.* Direct Measurement of Light Waves. *Science* **305**, 1267-1269 (2004).
5 Gagnon, J., Goulielmakis, E. & Yakovlev, V. S. The accurate FROG characterization of attosecond pulses from streaking measurements. *Appl. Phys. B* **92**, 25-32 (2008).
6 Eisele, M., Krüger, M., Schenk, M., Ziegler, A. & Hommelhoff, P. Production of sharp gold tips with high surface quality. *Rev. Sci. Instrum.* **82**, 026101 (2011).
7 Hüfner, S. *Photoelectron Spectroscopy: Principles and Applications*. (Springer, 2003).
8 Zherebtsov, S. *et al.* Attosecond imaging of XUV-induced atomic photoemission and Auger decay in strong laser fields. *J. Phys. B* **44**, 105601 (2011).
9 *FDTD Solutions, V8, Lumerical,* http://www.lumerical.com/tcad-products/fdtd/.
10 Johnson, P. B. & Christy, R. W. Optical Constants of the Noble Metals. *Phys. Rev. B* **6**, 4370-4379 (1972).
11 Skopalova, E. *et al.* Numerical simulation of attosecond nanoplasmonic streaking. *New J. Phys.* **13**, 083003 (2011).
12 Swope, W. C., Andersen, H. C., Berens, P. H. & Wilson, K. R. A computer simulation method for the calculation of equilibrium constants for the formation of physical clusters of molecules: Application to small water clusters. *J. Chem. Phys.* **76**, 637-649 (1982).
13 Kelkensberg, F., Koenderink, A. F. & Vrakking, M. J. J. Attosecond streaking in a nano-plasmonic field. *New J. Phys.* **14**, 093034 (2012).
14 Stockman, M. I. Nanofocusing of optical energy in tapered plasmonic waveguides. *Phys. Rev. Lett.* **93**, 137404 (2004).
15 Süßmann, F. & Kling, M. F. Attosecond nanoplasmonic streaking of localized fields near metal nanospheres. *Phys. Rev. B* **84**, 121406(R) (2011).
16 Smirnova, O., Spanner, M. & Ivanov, M. Y. Coulomb and polarization effects in laser-assisted XUV ionization. *J. Phys. B* **39**, S323-S339 (2006).
17 Nagele, S. *et al.* Time-resolved photoemission by attosecond streaking: extraction of time information. *Journal of Physics B: Atomic, Molecular and Optical Physics* **44**, 081001 (2011).
18 Thumm, U. *et al.* in *Handbook of Photonics* (ed David Andrews) Ch. 22, 537-553 (Wiley-Blackwell, 2015).
19 Dahlström, J. M., L'Huillier, A. & Maquet, A. Introduction to attosecond delays in photoionization. *J. Phys. B* **45**, 183001 (2012).
20 Pazourek, R., Nagele, S. & Burgdörfer, J. Time-resolved photoemission on the attosecond scale: opportunities and challenges. *Faraday Disc.* **163**, 353-376 (2013).
21 Schultze, M. *et al.* Delay in Photoemission. *Science* **328**, 1658-1662 (2010).
22 Smirnova, O., Mouritzen, A. S., Patchkovskii, S. & Ivanov, M. Y. Coulomb–laser coupling in laser-assisted photoionization and molecular tomography. *J. Phys. B* **40**, F197-F206 (2007).
23 Zhang, C. H. & Thumm, U. Electron-ion interaction effects in attosecond time-resolved photoelectron spectra. *Phys. Rev. A* **82**, 043405 (2010).
24 Baggesen, J. C. & Madsen, L. B. Polarization Effects in Attosecond Photoelectron Spectroscopy. *Phys. Rev. Lett.* **104**, 043602 (2010).
25 Dahlström, J. M. *et al.* Theory of attosecond delays in laser-assisted photoionization. *Chem. Phys.* **414**, 53-64 (2013).
26 Neppl, S. *et al.* Attosecond Time-Resolved Photoemission from Core and Valence States of Magnesium. *Phys. Rev. Lett.* **109**, 087401 (2012).
27 Neppl, S. *et al.* Direct observation of electron propagation and dielectric screening on the atomic length scale. *Nature* **517**, 342-346 (2015).

28 Cavalieri, A. L. *et al.* Attosecond spectroscopy in condensed matter. *Nature* **449**, 1029-1032 (2007).
29 Okell, W. A. *et al.* Temporal broadening of attosecond photoelectron wavepackets from solid surfaces. *Optica* **2**, 383-387 (2015).
30 Guénot, D. *et al.* Photoemission-time-delay measurements and calculations close to the 3s-ionization-cross-section minimum in Ar. *Phys. Rev. A* **85**, 053424 (2012).
31 Klünder, K. *et al.* Probing Single-Photon Ionization on the Attosecond Time Scale. *Phys. Rev. Lett.* **106**, 143002 (2011).
32 Kheifets, A. S. & Ivanov, I. A. Delay in Atomic Photoionization. *Phys. Rev. Lett.* **105**, 233002 (2010).
33 Moore, L. R., Lysaght, M. A., Parker, J. S., van der Hart, H. W. & Taylor, K. T. Time delay between photoemission from the 2p and 2s subshells of neon. *Phys. Rev. A* **84**, 061404 (2011).
34 Saha, S. *et al.* Relativistic effects in photoionization time delay near the Cooper minimum of noble-gas atoms. *Phys. Rev. A* **90**, 053406 (2014).
35 Guénot, D. *et al.* Measurements of relative photoemission time delays in noble gas atoms. *J. Phys. B* **47**, 245602 (2014).
36 Feist, J. *et al.* Time delays for attosecond streaking in photoionization of neon. *Phys. Rev. A* **89**, 033417 (2014).
37 Zhang, C. H. & Thumm, U. Attosecond Photoelectron Spectroscopy of Metal Surfaces. *Phys. Rev. Lett.* **102**, 123601 (2009).
38 Zhang, C. H. & Thumm, U. Probing dielectric-response effects with attosecond time-resolved streaked photoelectron spectroscopy of metal surfaces. *Phys. Rev. A* **84**, 063403 (2011).
39 Zhang, C. H. & Thumm, U. Streaking and Wigner time delays in photoemission from atoms and surfaces. *Phys. Rev. A* **84**, 033401 (2011).
40 Zhang, C. H. & Thumm, U. Effect of wave-function localization on the time delay in photoemission from surfaces. *Phys. Rev. A* **84**, 065403 (2011).
41 Lemell, C., Solleder, B., Tokesi, K. & Burgdörfer, J. Simulation of attosecond streaking of electrons emitted from a tungsten surface. *Phys. Rev. A* **79**, 062901 (2009).
42 Krasovskii, E. E. Attosecond spectroscopy of solids: streaking phase shift due to lattice scattering. *Phys. Rev. B* **84**, 195106 (2011).
43 Kazansky, A. K. & Echenique, P. M. One-electron model for the electronic response of metal surfaces to subfemtosecond photoexcitation. *Phys. Rev. Lett.* **102**, 177401 (2009).
44 Borisov, A. G., Sánchez-Portal, D., Kazansky, A. K. & Echenique, P. M. Resonant and nonresonant processes in attosecond streaking from metals. *Phys. Rev. B* **87**, 121110 (2013).
45 Novotny, L. & Hecht, B. *Principles of Nano-Optics*. (Cambridge University Press, 2006).
46 Pazourek, R., Nagele, S. & Burgdörfer, J. Probing time-ordering in two-photon double ionization of helium on the attosecond time scale. *J. Phys. B* **48**, 061002 (2015).
47 Locher, R. *et al.* Attosecond interferometry unravels complex delays in photoemission from solids. *Optica* **2**, 405-410 (2015).
48 Wu, L. & Ang, L. K. Nonequilibrium model of ultrafast laser-induced electron photofield emission from a dc-biased metallic surface. *Phys. Rev. B* **78**, 224112 (2008).
49 Pant, M. & Ang, L. K. Time-dependent quantum tunneling and nonequilibrium heating model for the generalized Einstein photoelectric effect. *Phys. Rev. B* **88**, 195434 (2013).
50 Yanagisawa, H. *et al.* Energy Distribution Curves of Ultrafast Laser-Induced Field Emission and Their Implications for Electron Dynamics. *Phys. Rev. Lett.* **107**, 087601 (2011).
51 Stockman, M. I., Kling, M. F., Kleineberg, U. & Krausz, F. Attosecond nanoplasmonic-field microscope. *Nat. Photon.* **1**, 539-544 (2007).
52 Süßmann, F. *Dissertation, LMU Munich (2013)*.
53 Herink, G., Solli, D. R., Gulde, M. & Ropers, C. Field-driven photoemission from nanostructures quenches the quiver motion. *Nature* **483**, 190-193 (2012).
54 Piglosiewicz, B. *et al.* Carrier-envelope phase effects on the strong-field photoemission of electrons from metallic nanostructures. *Nat. Photon.* **8**, 37-42 (2014).
